# Effectiveness of antimicrobial-coated central venous catheters for preventing catheter-related blood-stream infections with the implementation of bundles: a systematic review and network meta-analysis

**DOI:** 10.1186/s13613-018-0416-4

**Published:** 2018-06-15

**Authors:** Hongliang Wang, Hongshuang Tong, Haitao Liu, Yao Wang, Ruitao Wang, Hong Gao, Pulin Yu, Yanji Lv, Shuangshuang Chen, Guiyue Wang, Miao Liu, Yuhang Li, Kaijiang Yu, Changsong Wang

**Affiliations:** 1 Department of Critical Care Medicine, The Second Affiliated Hospital of Harbin Medical University, Harbin, China; 2 Department of Critical Care Medicine, Harbin Medical University Cancer Hospital, No. 150 Haping Rd., Nangang District, Harbin, 150081 China; 3 Department of Internal Medicine, Harbin Medical University Cancer Hospital, Harbin, China

**Keywords:** Central venous catheter, Catheter-related blood-stream infections, Catheter colonization, Bundles, Meta-analysis

## Abstract

**Background:**

Catheter-related blood-stream infections (CRBSIs) are the most common complication when using central venous catheters (CVCs). Whether coating CVCs under bundles could further reduce the incidence of CRBSIs is unclear. We aimed to assess the effectiveness of implementing the use of bundles with antimicrobial-coated CVCs for preventing catheter-related blood-stream infections.

**Methods:**

In this systematic review and network meta-analyses, we searched the Cochrane Central Register of Controlled Trials (CENTRAL) in the Cochrane Library in addition to the EMBASE, MEDLINE, CINAHL, and Web of Science databases for studies published before July 2017. The primary outcome was the rate of CRBSIs per 1000 catheter-days, and the secondary outcome was the incidence of catheter colonization.

**Results:**

Twenty-three studies revealed significant differences in the rate of CRBSIs per 1000 catheter-days between antimicrobial-impregnated and standard CVCs (RR 0.70, 95% CI 0.53–0.91, *p* = 0.008). Thirty-three trials were included containing 10,464 patients who received one of four types of CVCs. Compared with a standard catheter, chlorhexidine/silver sulfadiazine- and antibiotic-coated catheters were associated with lower numbers of CRBSIs per 1000 catheter-days (ORs and 95% CrIs: 0.64 (0.40–0.955) and 0.53 (0.25–0.95), respectively) and a lower incidence of catheter colonization (ORs and 95% CrIs: 0.44 (0.34–0.56) and 0.30 (0.20–0.46), respectively).

**Conclusions:**

Outcomes are superior for catheters impregnated with chlorhexidine/silver sulfadiazine or other antibiotics than for standard catheters in preventing CRBSIs and catheter colonization under bundles. Compared with silver ion-impregnated CVCs, chlorhexidine/silver sulfadiazine antiseptic catheters resulted in fewer cases of microbial colonization of the catheter but did not reduce CRBSIs.

**Electronic supplementary material:**

The online version of this article (10.1186/s13613-018-0416-4) contains supplementary material, which is available to authorized users.

## Background

Central venous catheters (CVCs) are an important component of the rescue and treatment of critically ill patients. CVCs have historically been extensively used in hemodynamic monitoring, tumor therapy, blood liquid purification, infusion and parenteral nutrition support. However, CVC use can cause a variety of complications. The most common complication is catheter-related blood-stream infections (CRBSIs) [1]. The International Nosocomial Infection Control Consortium (INICC) studied medical-surgical ICUs and found that the pooled rate of CRBSIs in China was 4.1/1000 central line-days, which was approximately 5 times higher than the rate of 0.8/1000 central line-days found in comparable US ICUs [2]. However, in 2631 cases across 7 ICUs, the estimated CRBSI rate was 7.66/1000 in August 2008 and July 2010 in four Chinese hospitals [3]. The cost per infection has been estimated to range from $34,508 to $56,000, and the annual cost of caring for patients with CRBSIs is $296 million to $2.3 billion in Spain [4]. Moreover, CRBSIs can increase the length of hospitalization and the amount of resources used and affect mortality.

In recent years, many approaches have been developed to reduce CRBSIs. The most promising approach is the use of central line bundles, which are recommended by the Institute for Healthcare Improvement as a means to improve patient care. Bundles include five components of care: hand hygiene, maximal barrier precautions, chlorhexidine skin antisepsis, optimal catheter site selection, and daily review of central line necessity. According to recent data, the implementation of central line bundles may reduce the rate of CRBSIs [5]. However, whether there are other ways to reduce CRBSIs has attracted the attention of scholars. Alternative methods include coating or impregnating catheters with anti-infective agents, including antibiotics, antiseptics, and antimetabolites. Moreover, CVCs that have been coated internally and/or externally with silver have also been shown to cause a lower incidence of catheter colonization and CRBSIs [6]. However, other authors have found the opposite results. It therefore remains controversial whether the incidence of CRBSIs is lower for coated catheters than for conventional standard catheters [7, 8]. The literature regarding various types of antimicrobial catheters and their effectiveness in preventing vascular-related blood-stream infections, especially with the implementation of bundles, remains sparse. Traditional meta-analyses can only compare two options that have been directly compared in head-to-head trials, and there is currently no consensus recommendation regarding which antimicrobial-coated CVC/bundle combination is best. Network meta-analyses (also called multiple or mixed treatment comparison meta-analyses, or MTCs) permit the evaluation of the comparative effectiveness of multiple interventions even when some pairs have not been directly compared and can potentially reduce uncertainty in treatment effect estimates [9, 10]. Given this background, our main aim was to evaluate the effectiveness of different types of antimicrobial-coated catheters in comparison with standard catheters as measured by the rate of CRBSIs per 1000 catheter-days among patients who received CVC and bundles. The incidence of catheter colonization was also studied as a secondary outcome. We hypothesized that antimicrobial-coated CVCs are more effective than standard catheters in preventing CRBSIs and catheter colonization in patients who received CVC and bundles.

## Methods

### Search strategy and selection criteria

We conducted a systematic review and network meta-analysis according to the Preferred Reporting Items for Systematic review and Meta-Analysis Protocol (PRISMA-P) [11]. This study was registered with the PROSPERO international prospective register of systematic reviews (number CRD42017055774).

RCTs were identified via electronic and manual searches. We searched the Cochrane Central Register of Controlled Trials (CENTRAL) in the Cochrane Library in addition to the CINAHL, EMBASE, MEDLINE, and Web of Science databases using a combination of Medical Subject Headings (MeSH) and text words (Additional file 1) for double-blind RCTs. We placed no restrictions on language or year of publication. The final search update was performed in July 2017. We also reviewed the reference lists of the identified meta-analyses. We manually searched the Index Medicus for RCTs, systematic reviews, and meta-analyses for supplemental literature that the initial electronic search missed. The literature screen was conducted by two separate groups. Any discrepancy was resolved by consensus. Literature search results were imported into EndNote X7 (Thomson Reuters, New York, USA) literature management software to exclude duplicate publications.

We included the following antimicrobial interventions regardless of their concentration: chlorhexidine/silver sulfadiazine, Oligon Vantex silver or silver, and other antibiotics, such as 5-fluorouracil, vancomycin, benzalkonium chloride, teicoplanin, miconazole/rifampicin, minocycline, and minocycline/rifampin. Trials involving patients who had received antimicrobial CVCs or standard non-impregnated polyurethane CVCs as well as implementation of a central line bundle containing at least one kind of the above antimicrobial interventions were included; however, RCTs that recruited participants without a control group or used quasi-random allocation were excluded.

### Data analysis

Two groups (YPL, CSS, LYJ, WGY, LM, and WY) independently extracted the data from each study. We used a self-designed standard template that included basic study information, such as title, author name, publication year, experimental time, country, characteristics of participants (e.g., total number of participants, age and gender), catheter characteristics (e.g., catheter position and type of catheter), intervention details (e.g., type and concentration of antimicrobials, days of catheter use), the specific process used in the trial, primary result variables (e.g., number of infections), and any additional preventive treatments that may have influenced outcomes (e.g., outcome measures and application of bundles). Any discrepancies were resolved by consensus by a review team (WCS, YPL, LYH, CSS, LYJ, GH, and WHL). The primary outcome of this study was the rate of CRBSIs per 1000 catheter-days. The implementation of a central line bundle containing at least one of the specified elements. If a study separately reported definite and probable CRBSIs, we did not include probable CRBSIs in blood-stream infections without laboratory confirmation. Our secondary outcome was catheter colonization, which was defined as the growth of more than 15 colony-forming units (CFUs) on the tip or subcutaneous segment of the catheter or positive semiquantitative results without clinical signs of sepsis.

### Statistical analysis

First, we performed a meta-analysis using the meta-R package (version 3.0.2) to compare antimicrobial-impregnated catheters with unimpregnated catheters. The meta-analysis was performed according to the PRISMA guidelines. Dichotomous data were analyzed using risk ratios (RRs) computed using the Mantel–Haenszel method (fixed or random models). Statistical significance was set at *p* < 0.05 for hypothesis testing. The Cochrane *Q* and *I*-square (*I*^2^) tests were used to evaluate the impact of study heterogeneity on the results of the meta-analysis. Statistical significance was set at 0.10 for Cochrane *Q* tests. Pooled results were constructed using either the fixed effect model in the absence of significant heterogeneity or the random effect model in the presence of significant heterogeneity [12]. We used the random effects model because it considers variation among studies and incorporates heterogeneity among studies in cases in which heterogeneity cannot be explained [13]. We assessed statistical heterogeneity with the *I*^2^ statistic using the Higgins–Thompson method (low heterogeneity, 25%; moderate, 50%; and high, 75%) [14]. When heterogeneity was present or suspected, we first checked that the original data for each study and the method used to extract the data were correct. If there was no problem, subgroup analyses or meta-regressions were conducted to explore the cause of the heterogeneity. We conducted exclusion sensitivity analyses to evaluate the contribution of individual studies to the global results. Sensitivity analyses excluding trials with high risk of bias (classified in any of the three aspects: randomized sequence generation, blinding and selective reporting) and trials that did not clearly specify patients included in the study are ICU patients were also performed. A funnel plot was used to detect potential publication bias [15].

Second, direct and indirect evidence for all included studies was combined in a network meta-analysis and estimated with maximum power. Network meta-analyses were performed within a Bayesian framework using the consistency model of the GeMTC package in R (i386 3.0.2) [16]. In this analysis, we calculated odds ratios (ORs) and 95% credibility interval (CrIs) to measure the effect of different antimicrobial CVCs on the rate of CRBSIs per 1000 catheter-days and the incidence of catheter colonization. CrIs were calculated using a Bayesian statistical method. Differences were considered statistically significant when the range of the 95% confidence interval did not include 1.0.

Model selection was performed based on the Dias guidelines for evaluating linear models. Dbar indicates the posterior mean of the residual deviance, pD indicates the effective number of parameters (leverage), and DIC indicates the “deviance information criterion”. Lower Dbar and DIC values indicate a better model fit. Differences between models of less than 3–5 were not considered significant. The models were run for 150,000 iterations, and convergence was assessed using the Brooks–Gelman–Rubin diagnostic. We used a technique known as “back calculation” to evaluate consistency in the findings of the network meta-analysis based on direct versus indirect evidence. During this process, three types of models were estimated: unrelated study effects, unrelated mean effects, and consistency.

The output of the summary function was plotted for visual representation. We investigated the possibility of statistical heterogeneity and inconsistency between direct and indirect effect estimates by visual inspection of the forest plots and the *I*^2^ statistic using the Higgins–Thompson method. We also ranked the different interventions in terms of their likelihood of leading to an association with the best results for each outcome. In a Markov chain Monte Carlo cycle, each antimicrobial CVC was ranked based on the estimated effect size. The sum of these probabilities is equal to 1 for each treatment and each rank. *X*% indicates that the strategy achieved *x*% effectiveness. Thus, a larger percentage indicates a more effective intervention. However, it represents only one possibility and does not indicate certainty.

## Results

**Fig. 1:**
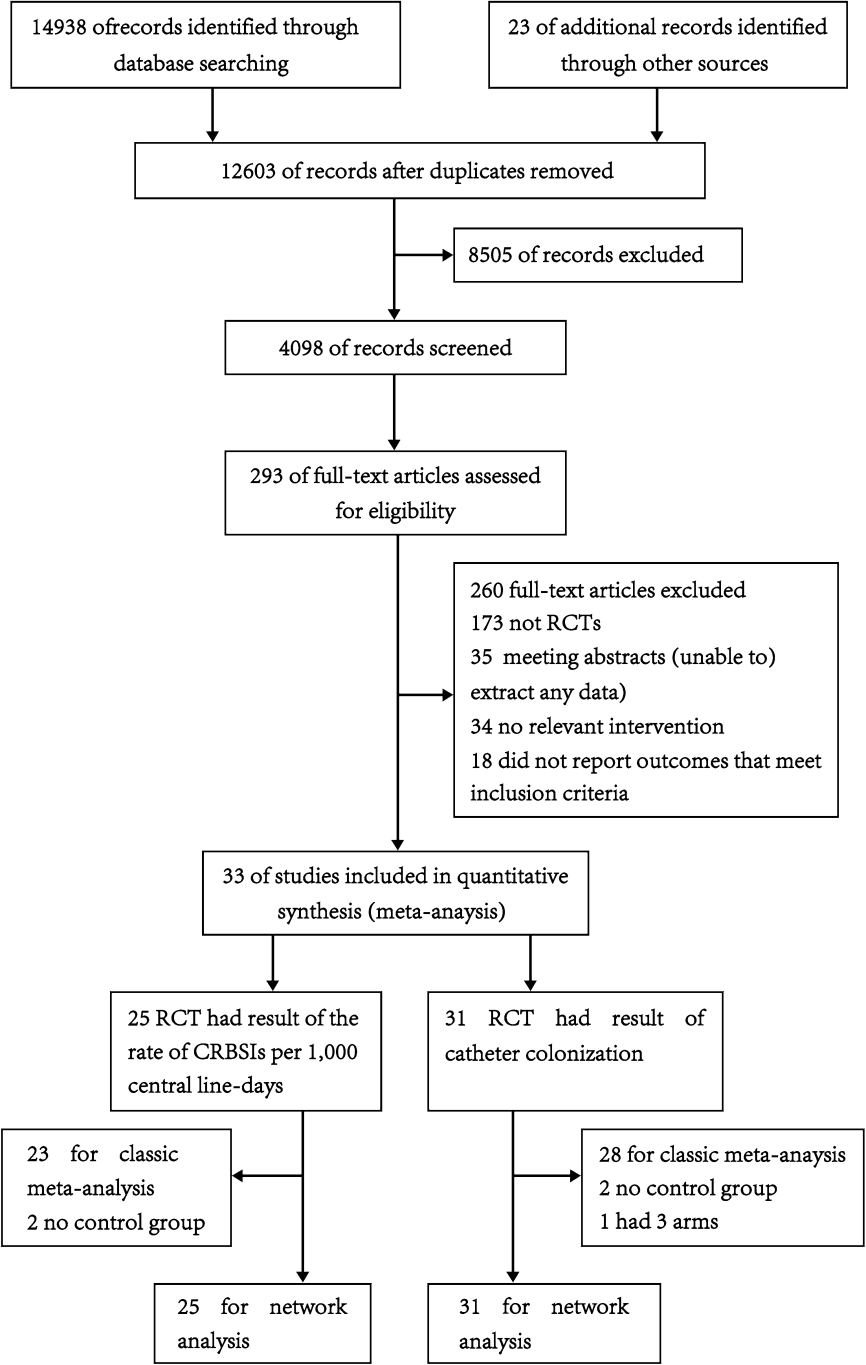
Flow diagram of literature search

We identified 14,938 studies by reviewing the titles and abstracts of the publications identified in the original search (Fig. 1). After this initial screening, we retrieved the full texts of potentially eligible articles for detailed assessment. Thirty-three RCTs [17–49] were included in the meta-analysis (Table 1). These studies included a total of 10,464 patients randomized to receive one of four types of CVCs.

**Table 1:** Characteristics of the randomized controlled trials

Study/year/Country	Antimicrobial-coated central venous catheters	Number of patients	Age (year) Mean ± SD	CRBSIs, *n*/total	Catheter duration, days	CR, *n*/total
Yücel et al. [17] /2004/Germany	AC versus CSC	223	62 (29–80)/61 (21–80)^ǂ^	0/118 0/105	6 (2–36)/6 (2–19)^†^	6/118 38/105
Walz et al. [18] /2010/USA	AC versus Chlo/SS	960	59.1 ± 15.6/60.1 ± 15.2	65/419 71/398	6.7 ± 4.8/6.8 ± 4.7	12/419 21/398
van Vliet et al. [19] /2011/The Netherlands	Chlo/SS versus CSC	94	67 ± 8/68 ± 7	Not reported	9 ± 7/10 ± 7	6/48 10/46
Thornton et al. [20] /1996/UK	AC versus OVS	176	Not reported	Not reported	Not reported	56/91 68/85
Theaker et al. [21] /2002/UK	Chlo/SS versus CSC	232	62.5	12/101 12/131	7.4/7.2	40/101 55/131
Tennenberg et al. [22] /1997/USA	Chlo/SS versus CSC	282	59.2 ± 1.1/57.9 ± 1.1	5/137 9/145	5.1 ± 0.2 5.3 ± 0.2	Not reported
Sheng et al. [23] /2000/China	Chlo/SS versus CSC	235	64 ± 18/61 ± 18	1/113 2/122	9.1 ± 5.5 8.2 ± 4.6	9/113 25/122
Moss et al. [24] /2000/UK	AC versus CSC	204	59/61	Not reported	3.79/4.25	21/106 38/98
Ostendorf et al. [25] /2005/Germany	Chlo/SS versus CSC	184	51/53*	3/90 7/94	12 (1–74)/10 (1–29)^†^	11/90 31/94
Osma et al. [26] /2006/Turkey	Chlo/SS versus CSC	133	49.4 ± 19.1/47.8 ± 17.5	4/64 1/69	11.7 ± 5.8 8.9 ± 4.6	14/64 14/69
Maki et al. [27] /1997/USA	CSC versus Chlo/SS	158	47 ± 18/49 ± 18	9/86 2/72	6.04 ± 3.41 5.96 ± 2.79	21/86 10/72
Logghe et al. [28] /2006/UK	OVS versus CSC	246	61/65	4/122 4/124	7 (3–21)/7 (2–18)^†^	71/122 91/124
León [29] /1997/Belgium	Chlo/SS versus CSC	680	51 ± 15.5/50 ± 15	17/338 15/342	20 + 12/20 + 13	Not reported
Khare et al. [30] /2004/Spain	AC versus CSC	367		6/187 11/180	10.3/10.4*	Not reported
Kalfon et al. [31] /2007/France	OVS versus CSC	617	61 ± 15/61 ± 17	8/320 8/297	10 (1–90)/10 (1–117)^†^	47/320 36/297
Jaeger et al. [32] /2005/Germany	Chlo/SS versus CSC	106	49/45*	1/51 8/55	14.3 ± 8.2/16.6 ± 9.7	5/51 59/55
Hagaua et al. [33] /2009/Romania	CSC versus OVS	272	55 ± 17/56 ± 20	4/131 8/141	9.7 ± 4.3/8.2 ± 4.1	30/131 38/14
Corral et al. [34] /2003/Spain	OVS versus CSC	145	56 ± 18/58 ± 18	1/80 4/65	14 ± 7/12 ± 7	29/80 41/65
Carrasco et al. [35] /2004/USA	Chlo/SS versus CSC	180	57 ± 16.9/55 ± 18.6	3/128 4/132	9.5/9*	13/128 29/132
Camargo et al. [36] /2009/Brazil	Chlo/SS versus CSC	109	73 (55–80)/74 (56–82)^†^	8/51 6/58	14 (7.5–21)/12 (8–19)^†^	15/51 20/58
Bun-Christian et al. [37] /2004/USA	Chlo/SS versus CSC	363	59.2 ± 17.8/58 ± 18.0	3/188 5/175	10.5 ± 8.9/12 ± 11.7	7/188 23/175
Bach et al. [38] /1999/Germany	OVS versus CSC	67	Not reported	2/34 2/33	Not reported	18/34 19/33
Bach et al. [39] /1996/Germany	AC versus CSC	20	Not reported	Not reported	Not reported	3/10 4/10
Antonelli et al. [40] /2012/Italy	OVS versus CSC	272	64.8 ± 16.6/62.9 ± 17.3	6/135 7/137	13 ± 24/15 ± 37	44/135 41/137
Moretti et al. [41] /2005/USA	OVS versus CSC	514		1/262 0/252	6.2/5.7*	24.4%/ 24.5%
Rupp et al. [42] /2005/USA	CSC versus Chlo/SS	777	61 ± 15.5/60 ± 16.4	3/393 1/384	5.13 (0.1–31.8)/142 (2–32.9)^†^	59/393 32/384
Rickard et al. [43] /2016/Australia	Chlo/SS versus CSC	404	55.06 ± 18.66/49.78 ± 19.79	1/203 0/201	5.11 ± 1.1/5.26 ± 1.23	10/189 19/186
Richards et al. [44] /2003/Australia	Chlo/SS versus CSC	460	58.3 ± 18.7/56.2 ± 20.3	2/237 6/223	8.4 ± 3.5/7.8 ± 3.2	14/237 30/223
Ranucci et al. [45] /2003/Italy	CSC versus OVS	545	65 ± 15.3/63.5 ± 15.2	12/277 9/268	9 ± 6.9/9.1 ± 7	31 ± 7/21 ± 4
Raad et al. [46] /1997/USA	CSC versus AC	298	56 (17–88)/58 (19–87)^†^	7/136 5/130	6 (1–21)/6 (1–28)^†^	36/136 11/130
Fraenkel et al. [47] /2006/Australia	AC versus OVS	646	53.2 ± 20.1/53.4 ± 19.5	4/280 5/294	6.23 ± 3.83/6.25 ± 3.9	25/280 43/294
Collin [48] /1998/USA	Chlo/SS versus CSC	220	46.4/47.2*	1/58 4/61	9.0 ± 6.1/7.3 ± 5.0	2/58 25/61
Dünser et al. [49] /2005/Australia	OVS versus Chlo/SS versus CSC	275	63 ± 16/62 ± 16/60 ± 16	Not reported	9.3 ± 4/9.7 ± 4/10.7 ± 4.2	27/160 12/165 19/160

*Chlo/SS* chlorhexidine/silver sulfadiazine, *OVS* Oligon Vantex silver, silver, *AC* antibiotic catheters: 5-fluorouracil, vancomycin, benzalkonium chloride, teicoplanin, miconazole/rifampicin, minocycline and minocycline/rifampin, *CSC* conventional standard catheter (single, double or triple-lumen, non-cuffed polyurethane catheters), *CRBSIs* catheter-related blood-stream infection, *CR* catheter colonization

Age and catheter duration presented as the mean (standard deviation), *median, ^†^median (range), or ^ǂ^mean (range)

### Risk of bias

We assessed the methodological quality of all eligible RCTs for each criterion as having a low, high, or unclear risk of bias using the Cochrane Collaboration’s risk of bias tool. Random sequence generation was adequately described in approximately half of the included trials (Additional file 2). In the remaining trials, the method of sequence generation was not specifically described. Allocation concealment and blinding were properly performed in approximately 13 and 12 of the 33 trials, respectively. Attrition bias and reporting bias were generally well reported in the included trials. The risk of bias assessed for each trial is presented in Additional file 2.

### Classic meta-analyses

#### The rate of CRBSIs per 1000 catheter-days

**Fig. 2:**
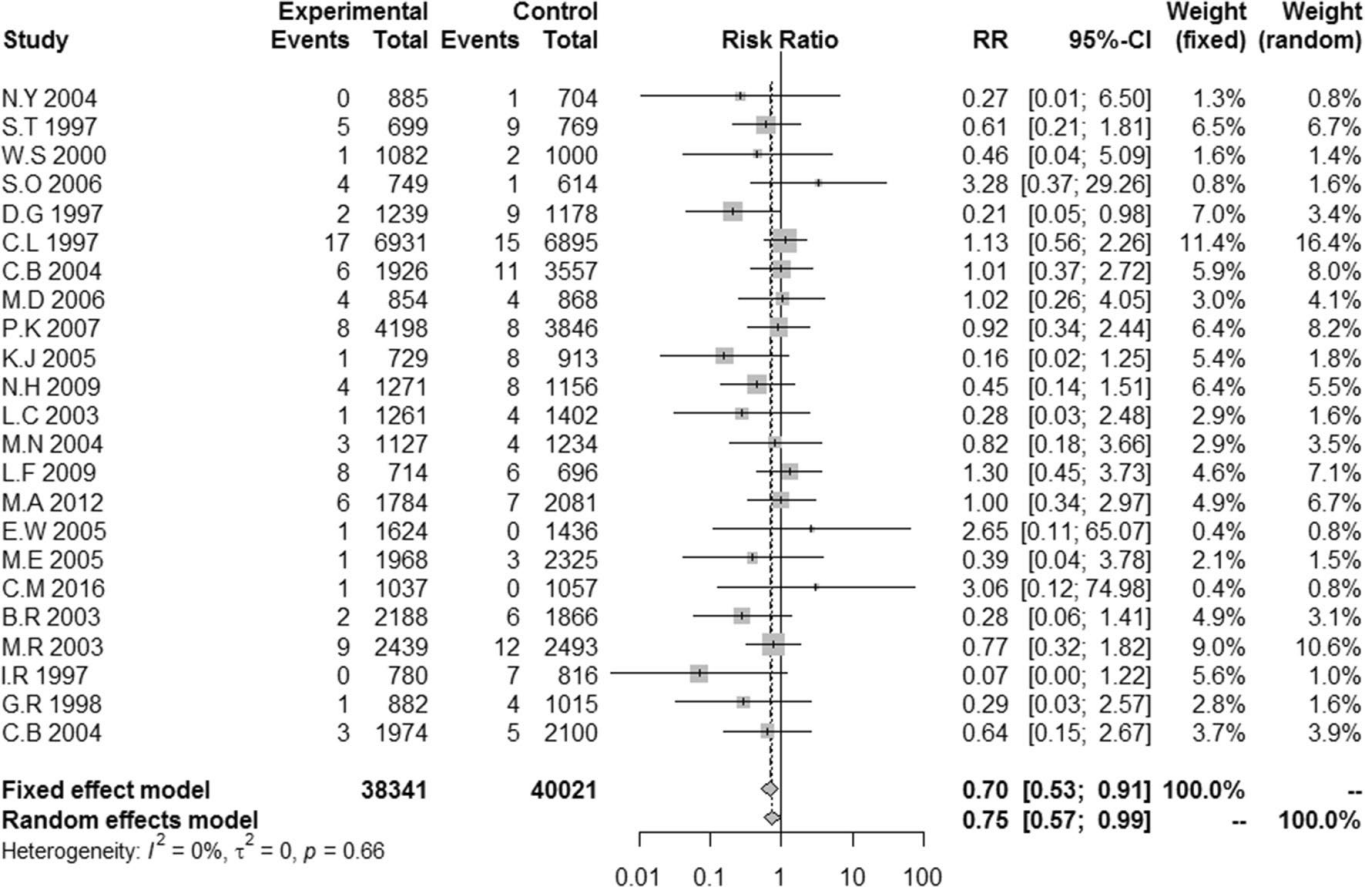
Relative risks for the rate of CRBSIs per 1000 catheter-days in the classic meta-analysis between antimicrobial-impregnated and standard non-impregnated CVCs

A meta-analysis of twenty-three studies revealed significant differences in the rate of CRBSIs per 1000 catheter-days between antimicrobial-impregnated and standard CVCs (RR 0.70, 95% CI 0.53–0.91, *p* = 0.008). A fixed model was used because no heterogeneity was noted among the studies (Cochran *Q* = 18.81, *p* = 0.66, *I*^2^ = 0.0%; Fig. 2).

#### Catheter colonization

A meta-analysis of twenty-eight studies revealed significant differences in catheter colonization between antimicrobial-impregnated and standard CVCs (RR 0.64, 95% CI 0.55–0.74, *p* < 0.0001). A random model was used as heterogeneity was noted among the studies (Cochran *Q* = 82.21, *p* < 0.0001, *I*^2^ = 67.2%) (Additional file 3). A funnel plot was used to assess publication bias in these studies (Additional file 4). No evident publication bias was observed in the visual distribution of the funnel plot. A sensitivity analysis showed that the stability of the results displayed no significant changes, validating the rationality and reliability of our analysis (Additional file 12).

### Network meta-analyses

#### The rate of CRBSIs per 1000 catheter-days

**Fig. 3:**
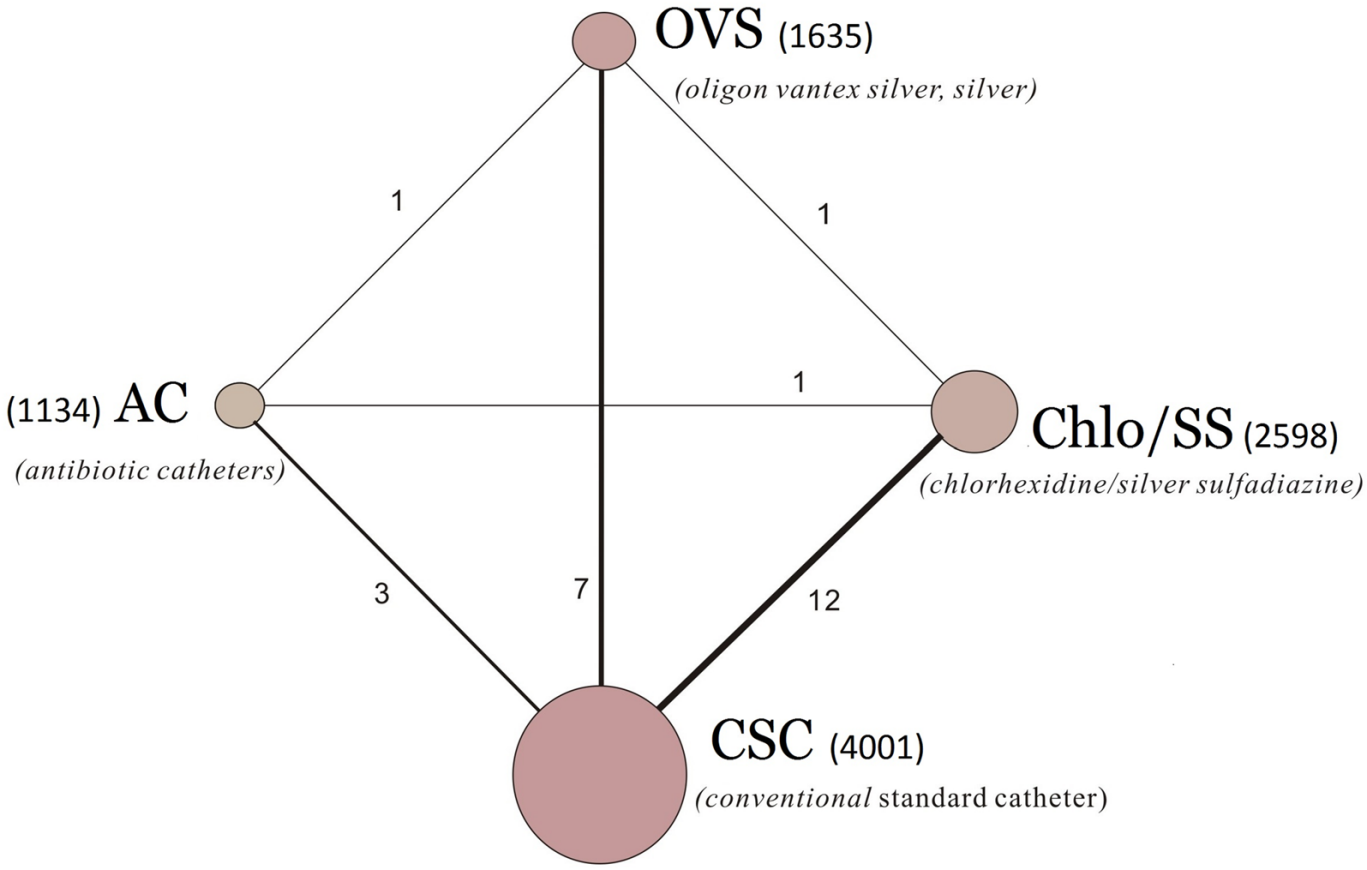
25 RCTs reported information on the rate of CRBSIs per 1000 central line-days

**Fig. 4:**
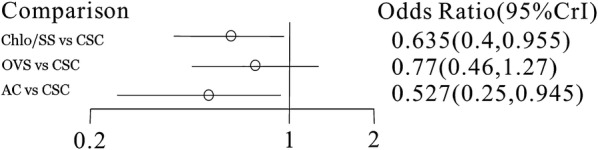
Numbers of CRBSIs per 1000 catheter-days: the conventional standard catheter versus chlorhexidine/silver sulfadiazine and antibiotic catheters

A total of 25 RCTs reported information on the rate of CRBSIs per 1000 central line-days and were included in the meta-analysis (Fig. 3). We chose a random effects model to evaluate the mean differences (MDs) in the overall effect sizes among the four types of CVCs studied (Additional file 5). All trials were two-arm randomized studies. Compared with the conventional standard catheter (single, double, or triple-lumen non-cuffed polyurethane catheters), chlorhexidine/silver sulfadiazine- and antibiotic-coated catheters (5-fluorouracil, vancomycin, benzalkonium chloride, teicoplanin, miconazole/rifampicin, minocycline, and minocycline/rifampin) were associated with lower numbers of CRBSIs per 1000 catheter-days (ORs and 95% CrIs: 0.64 (0.40–0.955) and 0.53 (0.25–0.95), respectively; Fig. 4). In addition, the OR and 95% CrI of Oligon Vantex silver or silver catheters were 0.77 (0.46–1.27), indicating the difference was not significant. All ORs and 95% CrIs for the antimicrobial CVC types studied in this report are shown in Additional file 6.

Most comparisons showed little or no heterogeneity. The endpoint of the *I*^2^ value for the rate of CRBSIs per 1000 catheter-days exceeded 50% (*I*^2^ = 68.2%) in three of the comparisons of type CSC CVCs (conventional, standard catheter) versus type AC CVCs (5-fluorouracil, vancomycin, benzalkonium chloride, teicoplanin, miconazole/rifampicin, minocycline, and minocycline/rifampin), indicating the presence of high heterogeneity (Additional file 6).

#### Catheter colonization

Thirty-one of the included studies reported the incidence of catheter colonization as a secondary outcome (Additional file 7). We chose a random effects model to evaluate the MDs in the overall effect sizes among the four types of CVCs studied (Additional file 8). Using the OR as the “combined effect size,” compared with the conventional standard catheter (single, double, or triple-lumen non-cuffed polyurethane catheters), chlorhexidine/silver sulfadiazine and antibiotic catheters (5-fluorouracil, vancomycin, benzalkonium chloride, teicoplanin, miconazole/rifampicin, minocycline, and minocycline/rifampin) were associated with lower incidences of catheter colonization (ORs and 95% CrIs: 0.44 (0.34–0.56) and 0.30 (0.20–0.46), respectively (Additional file 9). The ORs and 95% CrIs of other types of CVCs are shown in Additional file 10.

In Additional file 11, we summarize the rankings of the different competing types of CVCs according to the incidence of CRBSIs per 1000 catheter-days and in terms of catheter colonization, with details provided in Additional file 11. Antibiotic catheters (5-fluorouracil, vancomycin, benzalkonium chloride, teicoplanin, miconazole/rifampicin, minocycline, and minocycline/rifampin) had the greatest potential to reduce the incidence of CRBSIs per 1000 catheter-days, and the possibility of these catheters receiving the best ranking was 70.4%. The catheter type ranked second was chlorhexidine/silver sulfadiazine, which had a probability of 57.6%. Conventional standard catheters (single, double, or triple-lumen non-cuffed polyurethane catheters) ranked last. In terms of catheter colonization, other antibiotic catheters ranked lowest, with a probability of 94.4%. Conventional standard catheters had the highest probability of causing catheter colonization.

## Discussion

Our traditional meta-analysis showed that antimicrobial catheters were more effective than traditional catheters in preventing CRBSIs and catheter colonization when used in combination with bundles. A meta-analysis of twenty-three studies revealed significant differences in the rate of CRBSIs per 1000 catheter-days between antimicrobial-impregnated and standard CVCs (RR 0.70, 95% CI 0.53–0.91, *p* = 0.008). The results of a network meta-analysis demonstrated which antimicrobial catheters are most effective in adults. This review will aid in clinical decision-making. Our findings show that chlorhexidine/silver sulfadiazine and antibiotic catheters (5-fluorouracil, vancomycin, benzalkonium chloride, teicoplanin, miconazole/rifampicin, minocycline, and minocycline/rifampin) are associated with lower numbers of CRBSIs per 1000 catheter-days and a lower incidence of catheter colonization. The use of Oligon Vantex silver or silver did not provide statistically significant results.

The Institute for Healthcare Improvement has presented guidelines for the placement and care of CVCs. These guidelines were developed to reduce CRBSIs and involved the implementation of five components of care called the “central line bundle” [50]. A recent study by Erwin Ista indicated that implementation of central line insertion and maintenance bundles can potentially reduce the incidence of CRBSIs. In their meta-analysis, the incidence of infections decreased significantly from a median of 6.4/1000 catheter-days to 2.5/1000 catheter-days after the implementation of bundles [51]. However, no rigorous systematic review has previously compared the relative effectiveness of different antimicrobial-coated catheters when applied in combination with bundles.

A systematic review by Veenstra reported that using CVCs coated with a combination of chlorhexidine/silver sulfadiazine appears to effectively reduce the incidence of both catheter colonization and CRBSIs in patients. It is worth noting that chlorhexidine/silver sulfadiazine-coated catheters reduced the rates of CRBSI by approximately 40% and are therefore appropriate for use in patients at high risk of developing CRBSIs. This has particularly important clinical and economic significance for ICUs, in which the mortality of CRBSI is 10–35% [52]. Similarly, our results demonstrate that compared to silver ion-impregnated CVCs, this type of antiseptic catheter reduces microbial colonization but not CRBSIs. Chlorhexidine/silver sulfadiazine-impregnated catheters represent one of the most promising approaches for preventing CRBSIs because they can inhibit microbial membrane function and DNA replication [6]. Chlorhexidine and silver sulfadiazine have a synergistic effect. They are capable of altering bacterial cytoplasmic membrane permeability, causing bacterial cytoplasmic material extravasation. In vitro studies have shown that chlorhexidine/silver sulfadiazine-coated catheters exert an inhibitory effect on Gram-positive bacteria (e.g., *Staphylococcus epidermidis*, *S. aureus*, and *S. hemolyticus*), Gram-negative bacteria (e.g., *Pseudomonas aeruginosa*, *Escherichia coli*, *Enterococcus faecalis*, and *Klebsiella pneumoniae*), and yeasts (e.g., *Candida albicans*) [53–55]. However, the release of chlorhexidine/silver sulfadiazine from the catheter and the antimicrobial efficacy against *S. aureus* associated with its release is limited to 16 days from the date of commercial purchase. Hence, if CVCs are stored for long periods of time, no local antimicrobials will remain available for release when a biomaterial-associated infection occurs. Thus, further research should explore the potential for increasing the antimicrobial capacity of these catheters to longer time periods.

Silver CVCs include a number of catheter categories, such as silver platinum–carbon-impregnated, Oligon Vantex silver, and silver ion only catheters. Silver ions have a different mode of antibacterial activity and can bind to microbial DNA, thereby preventing bacterial replication, and to the sulfhydryl groups of metabolic enzymes, thereby deactivating them [56]. However, silver ion catheters were not more beneficial than the other types of catheters evaluated in this study in protecting CVCs against bacterial colonization and preventing CRBSIs in clinical settings. It is possible therefore that silver has little effect on CRBSI rates and catheter colonization. Although the results of individual studies are conflicting, a meta-analysis performed by Chen et al. [6] showed that silver-impregnated CVCs were not associated with reduced rates of bacterial colonization or CRBSIs.

Antiseptic or anti-adhesive catheter coatings, such as benzalkonium chloride, are cationic surfactants and non-oxidizing fungicides that can inhibit microbial membrane function and DNA replication. These coatings exert antimicrobial activity primarily against Gram-positive species, although at higher concentrations, they also exert effects against Gram-negative bacteria and Candida species. Minocycline/rifampin-coated catheters have been shown to increase the risk of fungemia and fungal catheter colonization [57]. However, minocycline/rifampin combination therapy, which has a broad antimicrobial spectrum of activity, was more effective than other antibiotics, including vancomycin and rifampin, at preventing *S. epidermidis* catheter colonization.

Certain limitations of our study should be considered. First, catheter materials can influence the incidence of CRBSIs. For example, the incidence of CRBSIs is lower for polytetrafluoroethylene and polyurethane catheters than for those made of polyvinyl chloride or polyethylene [58]. Second, we did not verify that the same antimicrobial concentration was used for catheters coated on both the internal and external surfaces. Third, the result of catheter colonization was not determined using standard semiquantitative cultures. These differences might be important factors that affected the differences observed in efficacy among various antimicrobial-impregnated catheters.

## Conclusions

In conclusion, our results demonstrate that antimicrobial-impregnated CVCs were more effective than standard non-impregnated CVCs in decreasing the rate of CRBSIs per 1000 catheter-days and catheter colonization with the application of bundles. Moreover, the capacities of catheters impregnated with chlorhexidine/silver sulfadiazine and other antibiotic catheters (5-fluorouracil, vancomycin, benzalkonium chloride, teicoplanin, miconazole/rifampicin, minocycline, and minocycline/rifampin) are superior to those of traditional catheters in preventing CRBSIs and catheter colonization when applied with bundles. Despite their demonstrated efficacy, we could not determine whether other antibiotic catheters are superior to chlorhexidine/silver sulfadiazine-impregnated catheters. Compared to silver ion-impregnated CVCs, chlorhexidine/silver sulfadiazine antiseptic catheters reduce microbial colonization but do not reduce CRBSIs.

## Additional files

**Additional file 1.** Search strategy.

**Additional file 2.** Random sequence generation of the included trials.

**Additional file 3.** Heterogeneity analysis by random model.

**Additional file 4.** Publication bias assessment.

**Additional file 5.** Model fit for the CRBSIs per 1000 catheter-days rate results.

**Additional file 6.** The CRBSIs per 1000 catheter-days rate estimates from a multiple treatment meta-analysis compared with the direct and indirect estimates, which were based on back-calculated, and pair-wise meta-analyses. Direct and indirect estimates of effect and the corresponding Bayesian “I2” for inconsistency were calculated. And the “I2” from pooled pair-wise meta-analysis for heterogeneity were also calculated.

**Additional file 7.** The incidence of catheter colonization.

**Additional file 8.** Model fit for catheter colonization rate results.

**Additional file 9.** The ORs and 95% CrIs: the conventional standard catheter versus chlorhexidine/silver sulfadiazine and antibiotic catheters.

**Additional file 10.** The catheter colonization rate estimates from a multiple treatment meta-analysis compared with the direct and indirect estimates, which were based on back-calculated, and pair-wise meta-analyses. Direct and indirect estimates of effect and the corresponding Bayesian “I2” for inconsistency were calculated. And the “I2” from pooled pair-wise meta-analysis for heterogeneity were also calculated.

**Additional file 11.** 1. The rankings of the different competing types of CVCs; 2. Rankings based on simulations in terms of the CRBSIs per 1000 catheter-days rate; 3. Rankings based on simulations in terms of catheter colonization rate.

**Additional file 12.** 1. Sensitivity analysis for the rate of CRBSIs per 1000 catheter-days to evaluate the contribution of individual studies to the global results; 2. Sensitivity analyses for the rate of CRBSIs per 1000 catheter-days; 3. Sensitivity analysis for catheter colonization to evaluate the contribution of individual studies to the global results; 4: Sensitivity Analyses for the rate of catheter colonization.

## Abbreviations

CRBSIs: catheter-related blood-stream infections
CVC: central venous catheter
CRI: catheter-related infection
CC: catheter colonization
RR: risk ratio
OR: odds ratio
CrI: credibility interval
