# Supplementary material for: Effectiveness of antimicrobial-coated central venous catheters for preventing catheter-related blood-stream infections with the implementation of bundles: a systematic review and network meta-analysis
Source: Ann Intensive Care. 2018 Jun 15;8:71. doi: 10.1186/s13613-018-0416-4 (PMC6002334; doi:10.1186/s13613-018-0416-4)
Supplement: Supplementary file 1 — Additional file 1. Search strategy. [file 13613_2018_416_MOESM1_ESM.docx]

**Additional file 1. Search strategy**

1. Search strategy for CENTRAL, The Cochrane Library

#1 MeSH descriptor: [Catheter-Related Infections] explode all trees

#2 MeSH descriptor: [Catheterization, Central Venous] explode all trees

#3 MeSH descriptor: [Catheters, Indwelling] explode all trees

#4 (central venous access OR central OR cv OR cvp OR subclavian vein OR umbilic*) or (central next venous next catheter*):ti,ab,kw

#5bloodstream infection' OR 'bloodstream infections' OR bsi:ti,ab,kw

#6 #1 or #2 or #3 or #4 or #5

#7 MeSH descriptor: [Intensive Care Units] explode all trees

#8 (intensive care or intensive care unit or critical care or critical illness or critically ill or critical* or ICU or nicu or picu or icus or nicus or picus):ti,ab,kw

#9 #7 or #8

#10 MeSH descriptor: [quality of health care] explode all trees

#11 (education* OR qualit* prevent* or control* or improv*):ti,ab,kw

#12(bundle* or program* or protocol* or checklist* or multidisciplin* or framework* or initiativ* or collaborat* or hygien* or quality control or optimal CVC site or CVC-kit or hand washing or infection control or disinfect* or skin decontamination or dressing or sponge or silver or silver sulfadiazine or antimicrobial lock solutions or ethanol lock therapy or antibiotic or antiseptic or antimicrobial or anticoagulants or chlorhexidine gluconate or nursing): ti,ab,kw

#13 #10 or #11 or#12

#14 #6 and #7 and #13

2. Search strategy for EMBASE (OvidSP)

1. Catheter-Related Infections / or Catheterization, Central Venous / or Catheters, Indwelling / or central venous access or central or cv or cvp or subclavian vein or

umbilic* or bloodstream infection' or 'bloodstream infections' or bsi.mp.

2. intensive care /or intensive care unit/ or critical care /or critical illness/ or critically ill/ or critical*/ or ICU/ or nicu /or picu/ or icus /or nicus/ or picus.mp.

3. quality of health care / or education* or qualit* or prevent* or control* or improv*/ or (bundle* or program* or protocol* or checklist* or multidisciplin* or framework* or initiativ* or collaborat* or hygien* or quality control or optimal CVC site or CVC-kit or hand washing or infection control or disinfect* or skin decontamination or dressing or sponge or silver or silver sulfadiazine or antimicrobial lock solutions or ethanol lock therapy or antibiotic or antiseptic or antimicrobial or anticoagulants or chlorhexidine gluconate or nursing).mp.

4. 1 and 2 and 3

5. (placebo.sh. or controlled study.ab. or random*.ti,ab. or trial*.ti,ab. or ((singl* or doubl* or trebl* or tripl*) adj3 (blind* or mask*)).ti,ab.) not (animals not (humans and animals)).sh.

6. 4 and 5

3. Search strategy for MEDLINE (OvidSP)

1. exp Catheter-Related Infections / or Catheterization, Central Venous / or Catheters, Indwelling / or central venous access OR central OR cv OR cvp OR subclavian vein OR umbilic* or bloodstream infection' or 'bloodstream infections' or bsi.mp.

2. exp intensive care /or intensive care unit/ or critical care /or critical illness/ or critically ill/ or critical*/ or ICU/ or nicu /or picu/ or icus /or nicus/ or picus.mp.

3. exp quality of health care / or education* or qualit*or prevent* or control* or improv*/ or (bundle* or program* or protocol* or checklist* or multidisciplin* or framework* or initiativ* or collaborat* or hygien* or quality control or optimal CVC site or CVC-kit or hand washing or infection control or disinfect* or skin decontamination or dressing or sponge or silver or silver sulfadiazine or antimicrobial lock solutions or ethanol lock therapy or antibiotic or antiseptic or antimicrobial or anticoagulants or chlorhexidine gluconate or nursing).mp.

4.1 and 2 and 3

5. ((randomized controlled trial or controlled clinical trial).pt. or randomized.ab. or placebo.ab. or clinical trials as topic.sh. or randomly.ab. or trial.ti.) not (animals not (humans and animals)).sh.

6. 4 and 5

4. Search strategy for CINAHL (EBSCOhost)

S1((MH“Catheter-Related Infections) OR (MH“Catheterization, Central Venous”) OR(MH“Catheters, Indwelling”) OR (central venous access OR central OR cv OR cvp OR'subclavian vein OR umbilic* or bloodstream infection' or 'bloodstream infections' or bsi)

S2 (MH “Intensive Care Units”) OR(intensive care or intensive care unit or critical care or critical illness or critically ill or critical* or ICU or nicu or picu or icus or nicus or picus)

S4 (MH “quality of health care”) OR (education* or qualit* or prevent* or control* or improv*) OR (bundle* or program* or protocol* or checklist* or multidisciplin* or framework* or initiativ* or collaborat* or hygien* or quality control or optimal CVC site or CVC-kit or hand washing or infection control or disinfect* or skin decontamination or dressing or sponge or silver or silver sulfadiazine or antimicrobial lock solutions or ethanol lock therapy or antibiotic or antiseptic or antimicrobial or anticoagulants or chlorhexidine gluconate or nursing)

S4 S1 and S2 and S3

5. Search strategy for ISIWeb of Science

#1 TS=Catheter-Related Infections or Catheterization, Central Venous or Catheters, Indwelling or central venous access OR central OR cv OR cvp OR subclavian vein OR umbilic* or bloodstream infection' or 'bloodstream infections' or bsi

#2 TS= Intensive Care Units or intensive care or intensive care unit or critical care or critical illness or critically ill or critical* or ICU or nicu or picu or icus or nicus or picus #3 TS= (quality of health care) OR (education* or qualit* or prevent* or control* or improv*) OR (bundle* or program* or protocol* or checklist* or multidisciplin* or framework* or initiativ* or collaborat* or hygien* or quality control or optimal CVC site or CVC-kit or hand washing or infection control or disinfect* or skin decontamination or dressing or sponge or silver or silver sulfadiazine or antimicrobial lock solutions or ethanol lock therapy or antibiotic or antiseptic or antimicrobial or anticoagulants or chlorhexidine gluconate or nursing)

#4 TS=(random* or placebo* or multicenter* or prospective) or TS=(trail* SAME (clinical or controlled))

#5 #1 and #2 and #3 and #4
