# Supplementary material for: Effectiveness of antimicrobial-coated central venous catheters for preventing catheter-related blood-stream infections with the implementation of bundles: a systematic review and network meta-analysis
Source: Ann Intensive Care. 2018 Jun 15;8:71. doi: 10.1186/s13613-018-0416-4 (PMC6002334; doi:10.1186/s13613-018-0416-4)

**Additional file 2. Random sequence generation of the included trials.**

**1. Risk of bias graph. Review authors' judgements about each risk of bias item presented as percentages across all included studies. Red=high risk; green=low risk; yellow=unclear**


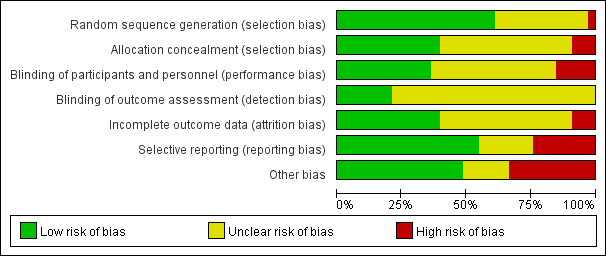


**2. Risk of bias summary (Red=high risk; yellow=unclear; green=low risk)**


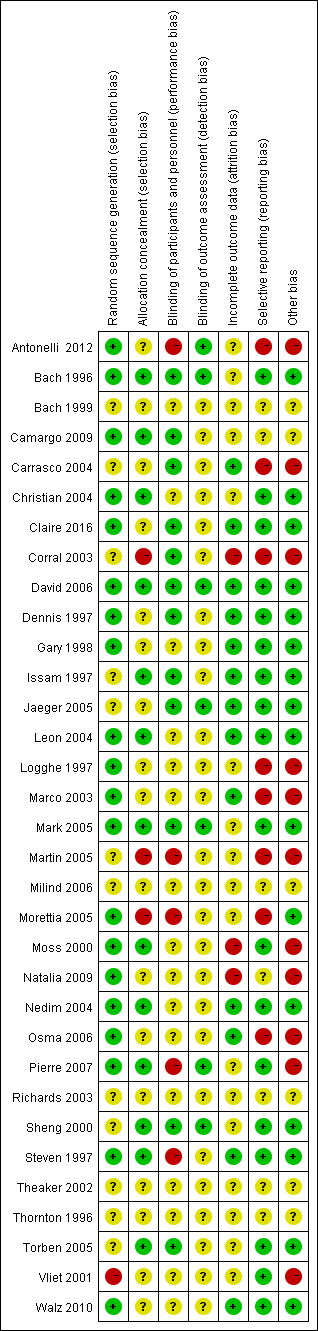

Supplement: Supplementary file 2 — Additional file 2. Random sequence generation of the included trials. [file 13613_2018_416_MOESM2_ESM.doc]
