# Supplementary figures and images for: Effectiveness of antimicrobial-coated central venous catheters for preventing catheter-related blood-stream infections with the implementation of bundles: a systematic review and network meta-analysis
Source: Ann Intensive Care. 2018 Jun 15;8:71. doi: 10.1186/s13613-018-0416-4 (PMC6002334; doi:10.1186/s13613-018-0416-4)

**Additional file 3.** **Heterogeneity analysis by random model**


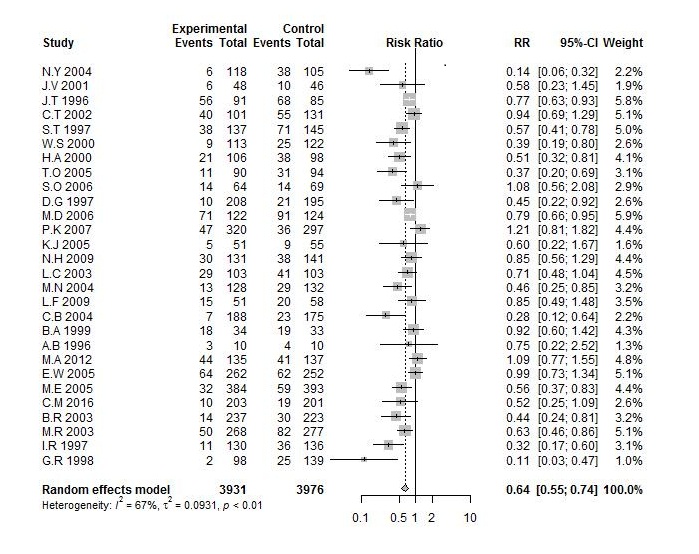

Supplement: Supplementary file 3 — Additional file 3. Heterogeneity analysis by random model. [file 13613_2018_416_MOESM3_ESM.doc]

**Additional file 4. Publication bias assessment**


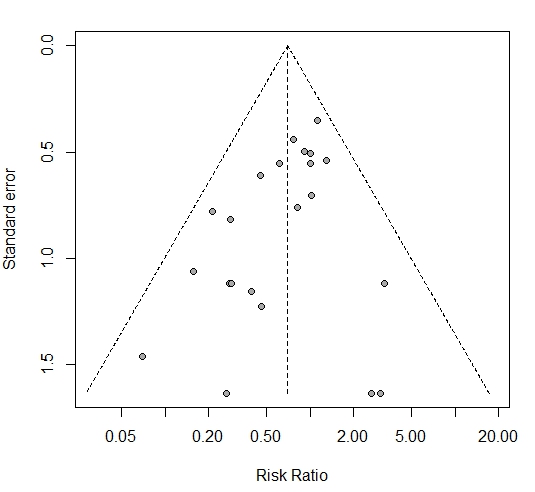


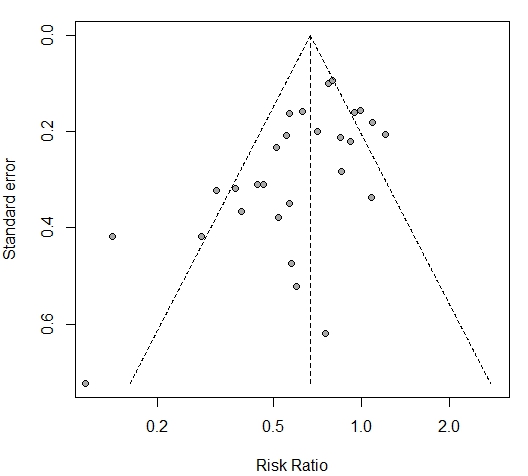

Supplement: Supplementary file 4 — Additional file 4. Publication bias assessment. [file 13613_2018_416_MOESM4_ESM.doc]
