# Supplementary material for: Effectiveness of antimicrobial-coated central venous catheters for preventing catheter-related blood-stream infections with the implementation of bundles: a systematic review and network meta-analysis
Source: Ann Intensive Care. 2018 Jun 15;8:71. doi: 10.1186/s13613-018-0416-4 (PMC6002334; doi:10.1186/s13613-018-0416-4)
Supplement: Supplementary file 5 — Additional file 5. Model fit for the CRBSIs per 1000 catheter-days rate results. [file 13613_2018_416_MOESM5_ESM.doc]

**Additional file 5. Model fit for the CRBSIs per 1000 catheter-days rate results**

|  | **Mean deviance** | **Penalty (pD)** | **DIC** |
| --- | --- | --- | --- |
| **Fixed effects model** | **450.06734** | **29.01799** | **479.08533** |
|
| **Random effects model** | **209.11532** | **32.36983** | **241.48514** |
|

Mean deviance indicates the posterior mean of the residual deviance. pD indicates the effective number of parameters (leverage).DIC indicates the ’Deviance Information Criterion’. A lower Mean deviance and DIC indicates a better model fit, based on the above information, random-effects model is the prefered model.
