# Supplementary material for: Effectiveness of antimicrobial-coated central venous catheters for preventing catheter-related blood-stream infections with the implementation of bundles: a systematic review and network meta-analysis
Source: Ann Intensive Care. 2018 Jun 15;8:71. doi: 10.1186/s13613-018-0416-4 (PMC6002334; doi:10.1186/s13613-018-0416-4)
Supplement: Supplementary file 7 — Additional file 7. The incidence of catheter colonization. [file 13613_2018_416_MOESM7_ESM.doc]

**Additional file 7. The incidence of catheter colonization**


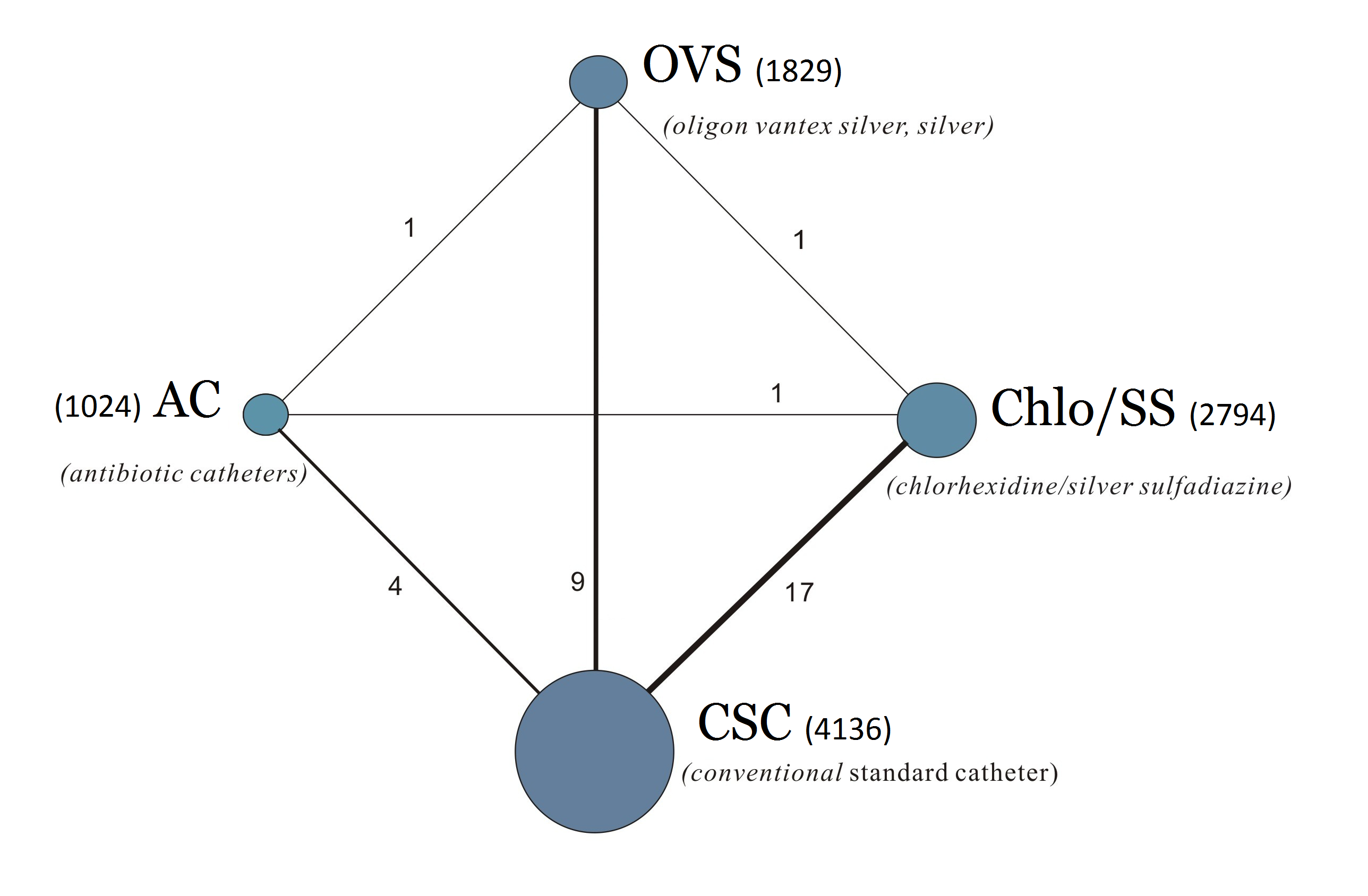


Abbreviations: Chlo/SS: chlorhexidine/silver sulfadiazine; OVS: oligon vantex silver, silver; AC: antibiotic catheters:5-fluorouracil, vancomycin, benzalkonium chloride, teicoplanin, miconazole/rifampicin, minocycline and minocycline/ rifampin; CSC: conventional standard catheter (single, double or triple-lumen, noncuffed polyurethane catheters)
