# Supplementary material for: Effectiveness of antimicrobial-coated central venous catheters for preventing catheter-related blood-stream infections with the implementation of bundles: a systematic review and network meta-analysis
Source: Ann Intensive Care. 2018 Jun 15;8:71. doi: 10.1186/s13613-018-0416-4 (PMC6002334; doi:10.1186/s13613-018-0416-4)
Supplement: Supplementary file 8 — Additional file 8. Model fit for catheter colonization rate results. [file 13613_2018_416_MOESM8_ESM.doc]

**Additional file 8. Model fit for catheter colonization rate results**

|  | **Mean deviance** | **Penalty (pD)** | **DIC** |
| --- | --- | --- | --- |
| **Fixed effects model** | **450.02648** | **28.98767** | **479.01415** |
|
| **Random effects model** | **357.21371** | **48.22607** | **405.43978** |
|
|

Mean deviance indicates the posterior mean of the residual deviance. pD indicates the effective number of parameters (leverage).DIC indicates the ’Deviance Information Criterion’. A lower Mean deviance and DIC indicates a better model fit, based on the above information, random-effects model is the prefered model.
