# Supplementary material for: Effectiveness of antimicrobial-coated central venous catheters for preventing catheter-related blood-stream infections with the implementation of bundles: a systematic review and network meta-analysis
Source: Ann Intensive Care. 2018 Jun 15;8:71. doi: 10.1186/s13613-018-0416-4 (PMC6002334; doi:10.1186/s13613-018-0416-4)
Supplement: Supplementary file 10 — Additional file 10. The catheter colonization rate estimates from a multiple treatment meta-analysis compared with the direct and indirect estimates, which were based on back-calculated, and pair-wise meta-analyses. Direct and indirect estimates of effect and the corresponding Bayesian “I2” for inconsistency were calculated. And the “I2” from pooled pair-wise meta-analysis for heterogeneity were also calculated. [file 13613_2018_416_MOESM10_ESM.doc]

**Additional file 10.** The catheter colonization rate estimates from a multiple treatment meta-analysis compared with the direct and indirect estimates, which were based on back-calculated, and pair-wise meta-analyses. Direct and indirect estimates of effect and the corresponding Bayesian ‘I^2^’ for inconsistency were calculated. And the‘I^2^’from Pooled pair-wise meta-analysis for heterogeneity were also calculated.


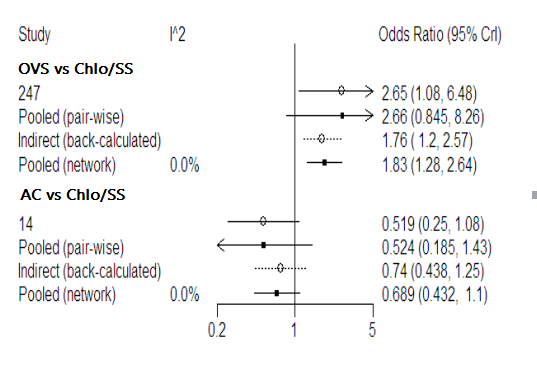


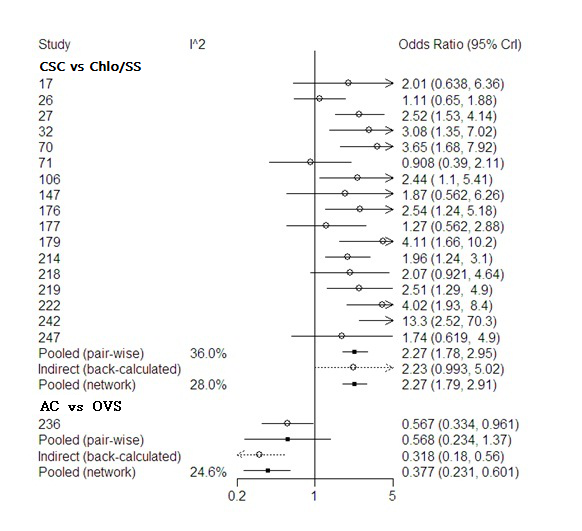


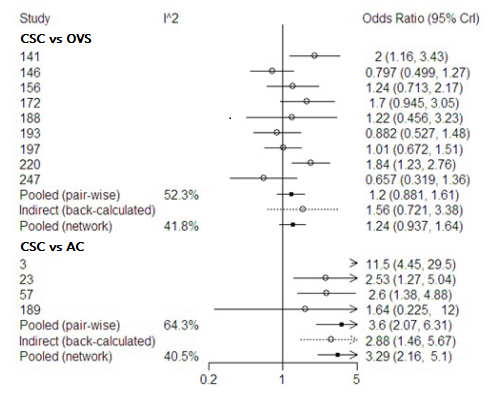


Abbreviations: Chlo/SS: chlorhexidine/silver sulfadiazine; OVS: oligon vantex silver, silver; AC: antibiotic catheters:5-fluorouracil, vancomycin, benzalkonium chloride, teicoplanin, miconazole/rifampicin, minocycline and minocycline/ rifampin; CSC: conventional standard catheter (single, double or triple-lumen, noncuffed polyurethane catheters)
