# Supplementary material for: Effectiveness of antimicrobial-coated central venous catheters for preventing catheter-related blood-stream infections with the implementation of bundles: a systematic review and network meta-analysis
Source: Ann Intensive Care. 2018 Jun 15;8:71. doi: 10.1186/s13613-018-0416-4 (PMC6002334; doi:10.1186/s13613-018-0416-4)
Supplement: Supplementary file 11 — Additional file 11. 1. The rankings of the different competing types of CVCs; 2. Rankings based on simulations in terms of the CRBSIs per 1000 catheter-days rate; 3. Rankings based on simulations in terms of catheter colonization rate. [file 13613_2018_416_MOESM11_ESM.doc]

**Additional file 11.**

1. **The rankings of the different competing types of CVCs.**


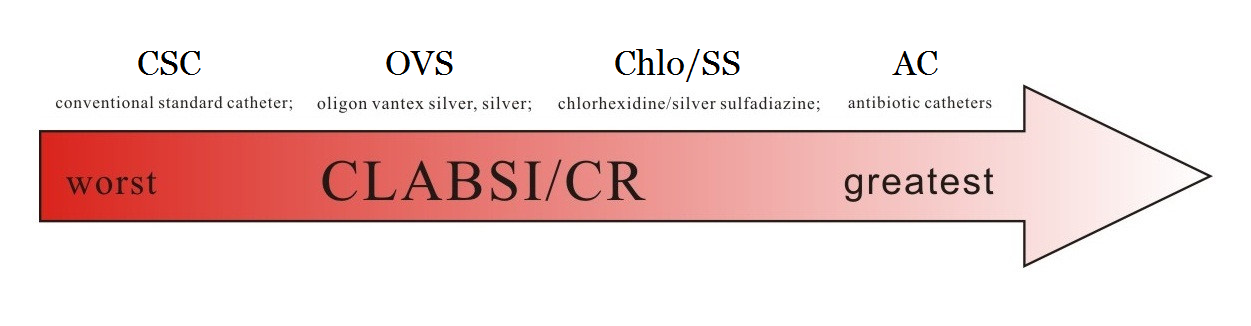


**2.** **Rankings based on simulations in terms of the CRBSIs per 1000 catheter-days rate.**

|  |  |  |  |  |
| --- | --- | --- | --- | --- |
|  | [,1] | [,2] | [,3] | [,4] |
| A | 1.09E-02 | 2.13E-01 | 5.76E-01 | 2.01E-01 |
| B | 14.35E-02 | 5.58E-01 | 2.03E-01 | 9.54E-02 |
| C | 1.28E-02 | 7.04E-02 | 2.13E-01 | 7.04E-01 |
| D | 8.33E-01 | 1.59E-01 | 8.16E-03 | 5.15E-04 |

The numbers in the table represent the probability that each treatment is highest (rank 1), the second highest (rank 2), etc. Rank probabilities sum to 1, both within a rank over treatments and within a treatment over ranks.

**3. Rankings based on simulations in terms of catheter colonization rate.**

|  |  |  |  |  |
| --- | --- | --- | --- | --- |
|  | [,1] | [,2] | [,3] | [,4] |
| A | 0 | 8.4E-04 | 9.43E-01 | 5.62E-02 |
| B | 6.21E-02 | 9.37E-01 | 9.65E-04 | 1.0E-05 |
| C | 0 | 1.35E-04 | 5.61E-02 | 9.44E-01 |
| D | 9.38E-01 | 6.21E-02 | 0 | 0 |

The numbers in the table represent the probability that each treatment is highest (rank 1), the second highest (rank 2), etc. Rank probabilities sum to 1, both within a rank over treatments and within a treatment over ranks.

Rank probability; preferred direction = 1
