# Supplementary material for: Effectiveness of antimicrobial-coated central venous catheters for preventing catheter-related blood-stream infections with the implementation of bundles: a systematic review and network meta-analysis
Source: Ann Intensive Care. 2018 Jun 15;8:71. doi: 10.1186/s13613-018-0416-4 (PMC6002334; doi:10.1186/s13613-018-0416-4)
Supplement: Supplementary file 12 — Additional file 12. 1. Sensitivity analysis for the rate of CRBSIs per 1000 catheter-days to evaluate the contribution of individual studies to the global results; 2. Sensitivity analyses for the rate of CRBSIs per 1000 catheter-days; 3. Sensitivity analysis for catheter colonization to evaluate the contribution of individual studies to the global results; 4: Sensitivity Analyses for the rate of catheter colonization. [file 13613_2018_416_MOESM12_ESM.docx]

**Additional file 12.**

**1:** **Sensitivity analysis for the rate of CRBSIs per 1,000 catheter-days to evaluate the contribution of individual studies to the global results**


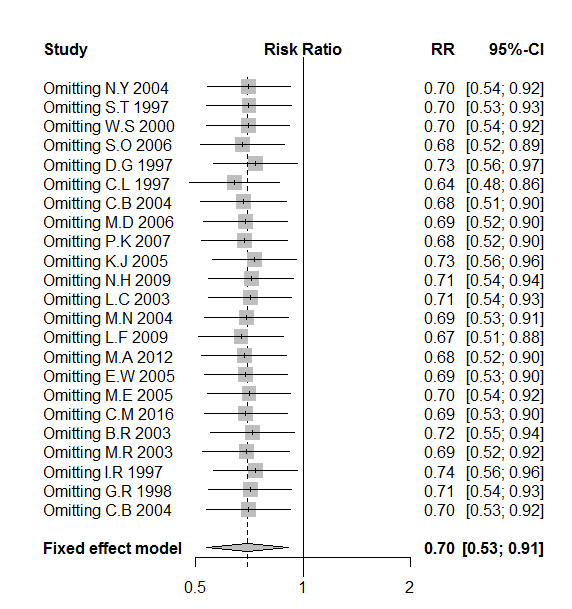


**2:**  **Sensitivity analyses for** **the rate of CRBSIs per 1,000 catheter-days**

Excluding trials with high risk of bias: Steven Tennenberg et al, S. Osma et al, C. Logghe et al, Pierre Kalfon et al, L. Corral et al, MN Carrasco et al, M. Antonelli et al, E.W. Moretti et al, Marco Ranucci et al.

Excluding trials that did not clearly specify patients included in the study are ICU patients: Nedim Yu cel et al, Steven Tennenberg et al, C. Logghe et al, K. Jaeger et al, E.W. Moretti et al, B. Richards et al, Marco Ranucci et al, Issam Raad et al.

| **the rate of CRBSIs per 1,000 catheter-days** | Antimicrobial-impregnated CVCs: events/total | Antimicrobial-impregnated CVCs: events/total | RR[95% CI] | Overall effect: *P* | Heterogeneity: *P*, *I^2^* |
| --- | --- | --- | --- | --- | --- |
| No exclusions (23 studies) | 88/38341 | 134/40021 | RR: 0.75[0.57; 0.99]  + FE: 0.70 [0.53, 0.91] | 0.04  0.008 | 0.66, 0.0%  0.66, 0.0% |
| Excluding trials with high risk of bias(14 studies) | 34/17529 | 74/19251 | RR: 0.58 [0.38; 0.89] | 0.01 | 0.49, 0.0% |
| Excluding trials that did not clearly specify patients included in the study are ICU patients  (15 studies) | 53/22066 | 76/24129 | RR: 0.78 [0.54; 1.13] | 0.19 | 0.77, 0.0% |

CRBSIs: Catheter-related blood-stream infections, CVC: central venous catheter, RR: relative risk, CI: confidence interval, ICU: intensive care unit

**3:** **Sensitivity analysis for catheter colonization to evaluate the contribution of individual studies to the global results.**


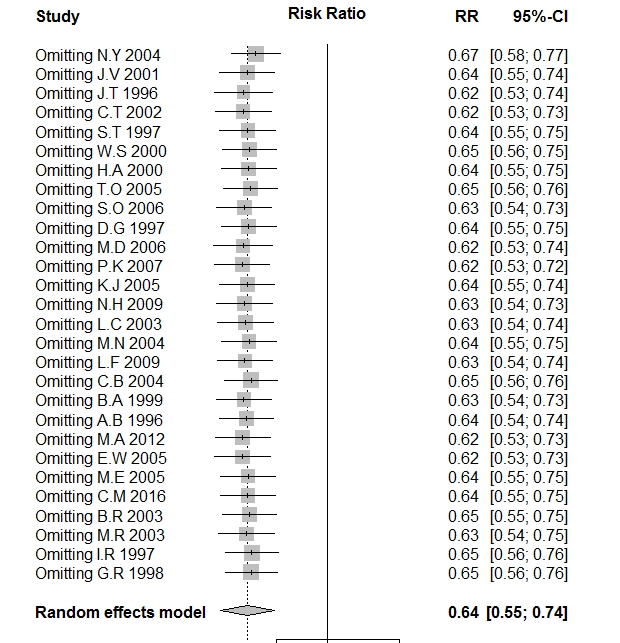


**4: Sensitivity Analyses for the rate of catheter colonization**

Excluding trials with high risk of bias: J van Vliet et al, Steven Tennenberg et al, S. Osma et al, Pierre Kalfon et al, L. Corral et al, MN Carrasco et al, M. Antonelli et al, E.W. Moretti et al, Marco Ranucci et al.

Excluding trials that did not clearly specify patients included in the study are ICU patients: Nedim Yu cel et al, Steven Tennenberg et al, H. A. Moss et al, Torben Ostendorf et al, K. Jaeger et al, E.W. Moretti et al, B. Richards et al, Marco Ranucci et al, Issam Raad et al.

| **the rate of catheter colonization** | Antimicrobial-impregnated CVCs: events/total | Antimicrobial-impregnated CVCs: events/total | RR[95% CI] | Overall effect: *P* | Heterogeneity: *P*, *I^2^* |
| --- | --- | --- | --- | --- | --- |
| No exclusions (28 studies) | 676/3931 | 1035/3976 | RR: 0.64[0.55; 0.74]  + FE: 0.67 [0.61; 0.72] | < 0.0001  < 0.0001 | < 0.0001, 67.2%  < 0.0001, 67.2% |
| Excluding trials with high risk of bias (19 studies) | 371/2466 | 649/2518 | RR: 0.55 [0.45; 0.68] | < 0.0001 | < 0.0001, 71.9% |
| Excluding trials that did not clearly specify patients included in the study are ICU patients  criteria (19 studies) | 456/2532 | 638/2591 | RR: 0.73 [0.63; 0.86] | < 0.0001 | 0.003, 53.6% |

CVC: central venous catheter, RR: relative risk, CI: confidence interval, ICU: intensive care unit
